# Diphtheria

**Published:** 1889-09

**Authors:** 


### DIPHTHERIA.

As illustrative of the deep importance attached to a study of the etiology, pathology and therapeutics of diphtheria, it may be mentioned that the King's County, N. Y., Medical Association discussed the subject "with interest and profit," says Dr. Squibbs, the President, "at six consecutive monthly meetings,"—and perhaps, then, did not fully exhaust it. It is one in which Texas physicians—as well as those of New York and elsewhere, are interested for the disease, in a malignant form, is unquestionably met with in this State; and we believe we cannot do better than to submit for their information a resume of the discussion, as relates to treatment, above alluded to by Dr. Squibb, in an editorial in the July number of his publication, the "Ephemeris."

Dr. Squibb says: "The importance of this discussion will be recognized from the fact that during the time occupied by it, no less than 594 deaths occurred from diphtheria in an estimated population of about 800,000; and from the circumstance that many of the best and busiest physicians of this population took part in the discussion. The monthly accounts of this discussion will be found in Gaillard's Medical Journal from November, 1888, to June, 1889, inclusive. All that is aimed at here is to give a brief abstract of the materia medica element of the discussion, so far as the principal agents used are concerned, and these agents will be taken up in the order of the frequency with which they are used in this locality. This, however, may not be the order of the real importance in treatment, because some have a popularity from long established use, which may be yielding to more recent agents. In almost all cases more than one of the agents were used.

"Alcohol was almost universally used, and generally as brandy or whisky, but with two distinct objects in view. The greater number of physicians used it in moderate quantities as a stimulant and a food to support the failing powers of the patients, and in the more exhausting stages of the disease, and always as an adjunct to other treatment. But there were many who used it in large quantities from the very onset of the disease, as a special, powerful antidote to the malignant poison of the disease very much in the way that is used in snake-bite. This treatment was pursued and strongly advocated by the late Dr. E. N. Chapman, of this city, up to the time of his death. Long before his death, however, he published a monogram on "The Antagonism of Alcohol and Diphtheria," Brooklyn, 1878, at which time he had treated 125 cases, with but one death. Many have followed this use of alcohol, as the principal element in treatment, with encouraging success, but not generally with the success of Dr. Chapman. One busy and successful physician, who had long used this treatment, during an epidemic in his practice, awoke one night at 3. a. m. with easily recognized symptoms of diphtheria, including a membrane in the pharynx. He at once took half a bottle of brandy,—probably about 12 fluidounces. This, under ordinary circumstance, would represent about 5.4 fluidounces of absolute alcohol, or about the fatal dose. By 8 o'clock,—or in five hours,—he had finished the bottle of, say, over 10 fluidounces of absolute alcohol. After such a use of alcohol he was "at work again in a week," and he believes that it saved his life. And yet when in health, half an ounce of brandy makes him feel "heady." Most of those who used alcohol in this way, testified to the absence of any signs of intoxication when the disease was at all pronounced in degree; and any signs of intoxication, or odor of alcohol on the breath were regarded as the limit of dosage. From 6 to 12 fluidounces of brandy in the twenty-four hours were not infrequently used on children from 6 months to 3 years old, though in the larger part of a general experience much smaller quantities were required;—but alcohol from the first in liberal and frequent doses.

"Tincture of the chloride of iron was perhaps the agent next most commonly used as a principal element in treatment, by the greatest number of practitioners, and this was always used freely as both a local and systemic agent. Though rarely used alone, there was much testimony in its favor, and none against it. It was simply losing ground to agents that were gaining in popularity.

"Corrosive sublimate, or mercuric chloride, was very largely used as a local application by spray or gargle,—about 1 to 500 for spraying, and 1 to 4,000 for gargling. A large number of physicians used this agent locally, and a smaller number used it systemically. In no case had there been toxic effects, and in one case, only, there was mild salivation, and the experience from its use was generally favorable. When used internally, the doses were generally small and frequently repeated, and the administration was carried to the production of characteristic diarrhœa with green stools. Attention was called to a recent paper on 'Mercuric Bi-Chloride in Diphtheria,' by Dr. John S. Coleman, of Augusta, Ga., published in the Journal of the American Medical Association for February 23, 1889, page 254. Dr. Coleman claims for mercuric chloride what has been, by others, claimed for alcohol,—that diphtheria establishes a tolerance of the agent, and certainly his experience tends to sustain such a claim. Among other cases he relates one of a child 16 months old who took corrosive sublimate in doses of one-eight of a grain hourly for seventy-two consecutive hours,—9 grains in three days,—with the result of complete recovery after expelling a large tube of laryngeal membrane. This was his largest dosage, and his worst case, but other children took very large quantities with similar results. A child of three years took six-eighths of a grain in six hours, and a total of 10.5 grains in nine days. Any such quantities in healthy children would probably prove fatal.

"Dr. Coleman does not always use such doses, but begins with one thirty-second of a grain, and increases until the disease is counteracted, or is in abeyance. It was stated by a member who knew him, that Dr. Coleman was not an enthusiast, but an old, long-established physician not likely to be carried away by having a theory to support.

"Peroxide of Hydrogen, or hydrogen dioxide, was presented during the discussion, as having been used both locally and internally in the treatment of diphtheria, as being one of the most active and energetic antiseptics known, and some account of it is given in this pamphlet at page 1146. It was subsequently used by several of the members, and the reports of its use then and since have been very favorable. Without being new at all, it is being treated as a novelty, and many very extravagant statements are being published by the makers of it, so that it is feared that this may prejudice the use of what should, on principle, be a very valuable agent, not only in diphtheria, but in many other septic conditions.

"As an ultimate result of the whole discussion it may be fairly stated that with the use of these agents, judiciously applied, the fatal cases of diphtheria, in the hands of those who discussed it here, were very far below its general mortality, and as this came to be apparent from the discussion it was questioned whether this was the result of treatment, or whether it might not be the result of a milder form of the disease in the classes of the community among which these physicians practiced. There was, however, no doubt left in the minds of any as to the value of treatment in contrast with expectant nursing of the sick,—and in this connection the writer offers no apology for quoting the weighty words of one who has observed diphtheria as closely, seen as much of it, and written upon it as intelligently as any living authority.

"Dr. A. Jacobi, of New York, in addressing the British Medical Association,—*The British Medical Journal* for September 22, 1888, page 653, says:

' "In urging upon you these remedial measures in the dangers of diphtheria, I beg of you not to take me for a meddler and interferer. Like yourself, I see many a case of sickness, and treat many without medicines. But there must be a reason for both non-medication and medication; and there must be medication when there is an indication for it. When there is, there is also an indication for a sufficient dose. I believe in Nature, and in spontaneous cures, but I know also that Nature destroys with the same equanimity and indifference with which it allows the getting well. I believe in the action of medicines, and the responsibility of the physician; I know that when a political Nihilist commits a single murder the newspapers of the world talk loudly of it. When a therapeutical Nihilist commits a hundred homicides, a quiet certificate and the silent sod cover the sins of omission." '

It is notorious that sufficient care is not excercised in making the diagnosis; nearly every form of tonsilitis, especially the follicular form, in which there is an exudation and deposit, is called diphtheria, and treated heroically. Fortunately, although diphtheria does occur here as elsewhere, it is comparatively rare,—but very fatal. We have had but limited experience in the treatment of this disease, but that experience led us long since to discard local cauterization, especially with nitrate of silver. More attention should be paid to nutrition and less to local treatment, in our judgment.

In the administration of alcohol the dosage should be regulated by the effects on the system, and not *so much* given in a given case, because there is authority for very large doses; there is such a thing as over-doing it, and the patient may be killed by alcohol as well as by septic poison. We distinctly remember a case of pneumonia in the person of an eminent physician, himself a believer in large doses of alcohol in all fevers. He was over-stimulated, and died of acute alcoholic poisoning!—In the above article we see a dangerous precedent, and warn our readers that twelve ounces of brandy in twenty-tour hours to a three year old will not do as a standard; there are sins of commission, as well as sins of omission, which a "certificate and the quiet sod" may cover up.

